# Role of muscle IL-6 in gender-specific metabolism in mice

**DOI:** 10.1371/journal.pone.0173675

**Published:** 2017-03-20

**Authors:** Amalia Molinero, Antonio Fernandez-Perez, Aina Mogas, Mercedes Giralt, Gemma Comes, Olaya Fernandez-Gayol, Mario Vallejo, Juan Hidalgo

**Affiliations:** 1 Institute of Neurosciences and Department of Cellular Biology, Physiology and Immunology, Faculty of Biosciences, Universitat Autònoma de Barcelona, Barcelona, Spain; 2 Centro de Investigación Biomédica en Red de Diabetes y Enfermedades metabólicas asociadas CIBERDEM, Instituto de Investigaciones Biomédicas Alberto Sols, Consejo Superior de Investigaciones Científicas/ Universidad Autónoma de Madrid (CSIC-UAM), Madrid, Spain; Universidade do Estado do Rio de Janeiro, BRAZIL

## Abstract

The aim of the present work was to further explore the physiological roles of muscle-derived IL-6. Adult-floxed and conditional skeletal muscle IL-6 knock out male and female mice were used to study energy expenditure (indirect calorimetry at rest and during treadmill exercise, and body temperature cycle during the light phase) and energy intake (response to fast/refeeding). We also evaluated the responses to leptin and the activity of the insulin signalling pathway in skeletal muscle and liver by phosphorylation of Akt at Ser 473. The stress response was also studied. Results indicate a relevant role of muscle IL-6 in maintaining energy homeostasis, especially in males. Absence of muscle IL-6 in male mice results in lower core body temperature in the light phase, increased respiratory exchange ratio (RER) both at rest and during exercise, increased expression of TCA cycle marked gene, citrate synthase in muscle, reduced fat storage and decreased body weight and food consumption in response to leptin. In females, muscle IL-6 deficiency increases VO_2_ and CO_2_ levels similarly. Also in contrast to males, energy expenditure (EE) measured over 48h reveals a significant elevation in female mice with muscle IL-6 deficiency; moreover, they show a modified response to fasting-refeeding and to restraint stress. The present results contribute to the understanding of the role of muscle IL-6 in male and female mouse metabolism, not only during exercise but also in the basal state and in situations where energy balance is altered.

## Introduction

Obesity represents one of the most serious health problems, resulting from an imbalance between energy intake and expenditure. Nutrients are used to obtain energy for metabolic basal rate, temperature homeostasis, and muscle function, whereas the excess of fuels is stored as fat. Energy expenditure is the counterpart of food intake, and body weight is stable when both energy expenditure and intake are in balance. The central nervous system (CNS) is a key player in both sensing and responding to changes in energy flux. To maintain balance or homeostasis, the CNS senses fuel availability and generates a response to the neural circuits that control food intake and energy expenditure. Basal metabolism, thermogenesis, and physical activity are each controlled by different pathways and effector organs [1–3].

The muscle is considered to be a major tissue for the disposal of both glucose [4] and fatty acids [5]. In addition to its obvious roles in motility, skeletal muscle plays a central role in the control of whole-body metabolism. Interleukin-6 (IL-6) can be classified as a myokine when its origin is the skeletal muscle; it is upregulated and secreted into the bloodstream in response to muscle contraction [6]. Putative roles of IL-6 in the adaptations of muscle associated with fasting or exercise have been extensively studied [6–8].

It is becoming increasingly recognized that obesity results in a chronic low grade inflammation accompanied by increased production of cytokines such as IL-6 [9,10]. In this way the circulating IL-6 level correlates with adipose tissue mass [9]. Several studies demonstrated that systemic- IL-6 KO mice develop mature-onset obesity [11,12] whereas adeno-associated viral delivery of IL-6 in rat hypothalamus [13] or central over-expression of IL-6 [14,15] decreased fat content and body weight gain. These results strongly suggest that IL-6 is involved in the control of body weight. Moreover, IL-6 could cause these effects through regulation of hypothalamic neuropeptides involved in energy homeostasis [15–17].

Circulating IL-6 may have access to the CNS [18] to regulate energy balance, but it may also affect peripheral tissues [7,19]. How peripheral IL-6 is involved in body weight regulation remains to be clearly established. To give some insight into this problem, we have produced floxed mice for IL-6 [20] and deleted its expression specifically in skeletal muscle using *myosin light chain 1f(Mlc)-Cre* mice [21]. In previous studies we demonstrated that muscle IL-6 deficiency did not cause major morphological alterations of skeletal muscle fibers [22]. In contrast, it did have a role in the control of body weight and body fat in a gender specific manner despite no evidence of an alteration in food intake [23]. Presumably, alteration of body weight without concomitant changes in food intake is due to altered energy expenditure [24]. The aim of the present work was to expand previous results exploring how muscle-derived IL-6 influences these two aspects of energy metabolism, and if phenotypical differences exist between sexes. In order to do so we used indirect calorimetry at rest and during treadmill exercise, and measured body temperature in the light phase. In addition, we evaluated the response to fast/refeeding and leptin administration. Finally, in order to determine whether the results were affected by a stress response or by changes in insulin response, we evaluated the pituitary-adrenal response to stress and the muscle and liver insulin-signalling pathway.

## Material and methods

### Animals

**Fig 1:**
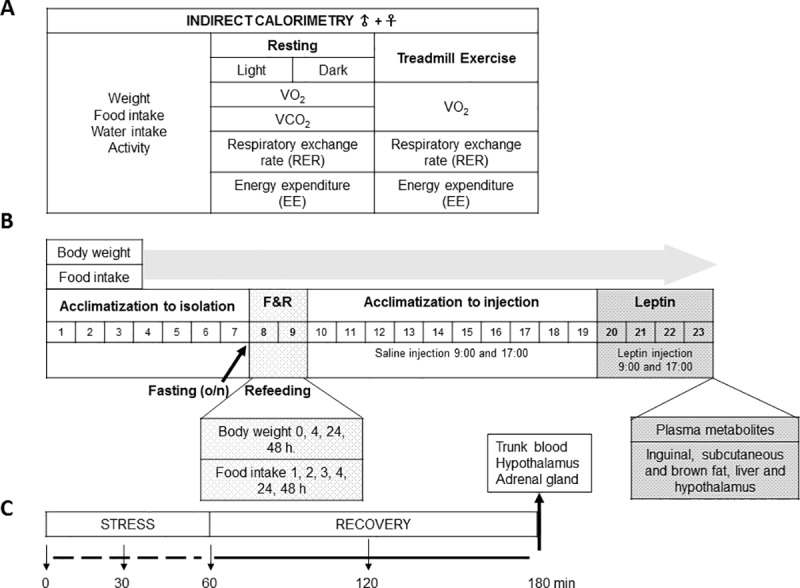
Experimental designs. **A:** Indirect calorimetry parameters (VO_2_, VCO_2,_ RER, and EE) in male and female *muscle IL-6 KO* (7/8) and floxed (8/8) mice, were analysed in resting (light and dark phases) and in treadmill exercise conditions. Weight, food and water intake and activity were controlled daily. **B**: Floxed and *muscle IL-6 KO* 3–4 month-old male (n = 13/12) and female (n = 14/14) mice were used for fasting and refeeding (F&R) and after recovery for leptin response studies. Several days of acclimatization to isolation and twice daily injection were required. Body weight and food intake were registered daily throughout the whole period (23 days), and specifically (0, 4, 24 and 48h; and 1, 2, 3, 4, 24 and 48h, respectively) when it was necessary for the experimental conditions (F&R). **C:** Floxed and *muscle IL-6 KO* 5–6 month-old male (n = 11/10) and female (9/10) mice were subjected to 60 minutes of restraint stress and then recovered for 120 minutes, after which they were euthanized and the trunk blood obtained. Tail blood was obtained at 0, 30 and 60 min of restraint stress.

Muscle-specific IL-6 KO mice (*muscle IL-6 KO*) were generated as described previously [23]. Briefly, floxed IL-6 mice [20], with further (>10) backcrossing with C57Bl/6 mice, were crossed with *myosin light chain 1f(Mlc)-Cre* mice, generously provided by Dr. Steve Burden [21]. The progeny was genotyped by PCR analysis of DNA extracted from tails. Muscle IL-6 mRNA levels were significantly decreased in *muscle IL-6 KO* mice compared to floxed mice [22,23]. Male and female mice were kept under constant temperature and standard 12-h light/12-h dark cycle. All mice received standard chow diet (Global Diet 2018, Teklad, Envigo) with free access to food and water unless otherwise stated. A general overview of some of the experiments carried out is shown in Fig 1.

### Ethical approval

Ethical approval for the use and experimentation of all mice in this study was obtained from Animal and Human experimental Ethics Committee of the Universitat Autonoma de Barcelona in agreement with the ethical and legal requirements (5/1995/Generalitat de Catalunya, 214/1997/Generalitat de Catalunya, Real decreto 1201/2005, 86/609/CEE, 91/628 and 92/65/UE; DAAM: n° 4522) following the “Principles of Laboratory Animal Care,” (2010). The animals were manipulated by authorized experimenters and daily monitored to minimize suffering and stress. No administration of analgesics was necessary.

### Energy expenditure experimental procedures

#### Indirect calorimetry, locomotor activity and food intake analyses

Three to four-month-old *muscle IL-6 KO* and floxed male (n = 7/8) and female (n = 8/8) mice were studied separately after transport to Madrid’s calorimetric laboratory (Fig 1A).

Indirect calorimetry analyses were carried out using a 16-chamber TSE PhenoMaster monitoring system (TSE Systems GmbH, Bad Homburg, Germany). Mice were moved into the room where the apparatus was located 4 days before measurements, and were placed in individual measuring cages for at least 24 h prior to the beginning of the experiment. Full access to food and water was continuously available, and their intake was monitored using built-in devices located within each cage. Calorimetry measurements were carried out during a period of 48 h, according to animal weight, to exclude that changes in body weight would contribute to differences in energy expenditure measurements [25]. The mice were on a 12- hour light-dark cycle (lights on at 08:00) and room temperature was maintained at 22 ± 2°C. Oxygen consumption and CO_2_ release were measured. From these values, respiratory exchange ratio (RER) was determined as VCO_2_/VO_2_ and energy expenditure was calculated as EE = (3.185 + 1.232 x RER) x VO_2_. Total locomotor activity (ambulatory and fine) was simultaneously measured on the X and Y axes using an infrared photocell beam interruption method.

#### Indirect calorimetry during acute exercise

Oxygen consumption and CO_2_ release during an acute running exercise were measured in a closed one-lane treadmill chamber tilted at an angle of 12.5° from horizontal, using a TSE PhenoMaster monitoring system (TSE Systems GmbH, Bad Homburg, Germany) (Fig 1A). Each mouse was individually placed in the treadmill chamber for 5 min for equilibration of gas before measurements were taken. After this, two basal gas measurements were taken at one-minute intervals. The treadmill speed was initially set at 0.15 m/s for 2 minutes, and then increased at 0.2 m/s and maintained constant for a further two-minute period. After this, acceleration was introduced, so that speed was increased by 0.05 m/s every two minutes. Mice were encouraged to run by delivering a small electric discharge (1.0 mA) from an electrode located at the lower end of the treadmill. The elapsed time to the first shock and the distance run were also recorded. A cut-off time of 22 min equivalent to a speed of 0.65 m/s was established. All mice tested kept running throughout the duration of the experiment.

#### Temperature cycle

Temperature was measured by a lubricated rectal probe (Cibertec) during the light phase at 7, 11, 15 and 19 hours in 5-6-month-old floxed and *muscle-IL6 KO* male (weight = 30.7 ±2.5 g and 30.27 ± 2.9 g, respectively; n = 11/10) and female (weight = 23.0 ±1.5 g and 23.6 ± 1.7 g, respectively; n = 9/10) mice.

### Energy intake experimental procedures

Floxed and *muscle-IL6 KO* 3–4 month-old male (n = 13/12) and female (n = 14/14) mice were used for energy intake studies (Fig 1B). In order to study the response to fasting and refeeding and to leptin treatment mice were individually housed. Because of this, adaptation of the animals to isolation was first monitored daily for 7–8 days (males and females, respectively) by evaluation of food intake and weight of mice.

#### Fasting and refeeding

After the 7–8 days of isolation, mice were fasted overnight (12–14 h), and the next morning glycaemia and body weight were recorded, and refeeding initiated (Fig 1B, left). Food consumption was tested 1, 2, 3, 4, 24, and 48 h after refeeding, and body weight was measured 4, 24 and 48 h after refeeding. Glycaemia was measured in tail samples with an ACCU CHECK glucometer (Roche) at the end of fasting and 4 h after refeeding.

#### Leptin treatment

In order to determine the role of muscle IL-6 deficiency in a situation similar to high level of energy storage, injection of leptin was carried out with the same animals used in the fasting and refeeding experiment after their body weight had recovered (Fig 1B, right). To better control the effect of leptin, animals were previously adapted to the injection procedure for 10 days. Adaptation included two intraperitoneal (i.p.) saline injections at 9:30 and 17:00 h; body weight and food intake were measured during this adaptation period. After the 10th day of saline injections, the two daily injections of saline were replaced with two injections of 120 μg of recombinant human leptin (Peprotech) or vehicle (PBS 0.1% BSA) (i.p). Body weight and food consumption were recorded daily.

On the 4^th^ day, in the morning, 3 hours after the leptin/vehicle injection, animals were weighed and euthanized by decapitation. Blood was collected from the trunk and allowed to clot for obtaining the serum, where several metabolites were analysed. The brain was removed, and the hypothalamus dissected and snap-frozen in liquid nitrogen for RT-qPCR. Liver, visceral (gonadal) and subcutaneous (inguinal region) white adipose tissue (WAT), and interscapular brown adipose tissue (BAT) were removed and weighed. Tibialis anterior, WAT and BAT were snap-frozen in liquid nitrogen for RT-qPCR analysis.

### Insulin signalling pathway

The effects of muscle IL-6 deficiency on body weight, fat regulation and tolerance to insulin administration have been previously shown [23]. To further analyse the effects of muscle IL-6 absence, the activation of Akt after 30 min after insulin administration (i.p. 1 UI/animal) in mice starved for 4 h was studied in two muscle types, soleus (mostly oxidative) and tibialis anterior (mostly glycolytic) and liver by western blot. Insulin or vehicle was injected in *muscle IL-6 KO* (n = 8/6) and floxed (n = 7/5) male and *muscle IL-6 KO* (n = 8/7) and floxed (n = 7/6) female mice. In order to compare all samples, several repeated samples were loaded in all gels, and the gel-to-gel variation coefficient was calculated and applied.

### Stress response

Because it is considered that experimental procedures that are associated with isolation or injection (such as fasting-refeeding and leptin treatment) may trigger a stress response, we performed acclimatization to these procedures prior to the experiments (Fig 1B). Since we observed differences between genotypes and sexes during these acclimatization periods, we designed an experiment to analyze the stress response by studying the Hypothalamus-Pituitary-Adrenal axis (HPA) (Fig 1C). Restraint stress was carried out in a 50-ml ventilated conical tube for 60 min [26]. Floxed and *muscle-IL6 KO* 5–6 month-old male (n = 11/10) and female (9/10) mice were subjected to restraint stress. Tail blood was collected at 0, 30 and 60 min during stress time and after 120 min of recovery time (180 min), after which mice were euthanized by decapitation. The adrenal glands were removed, weighed and stored (-80°C), and the hypothalamus was dissected, snap-frozen in liquid nitrogen, and stored at -80°C for RT-qPCR. Plasma corticosterone levels were determined by radioimmunoassay (RIA). Total adrenal protein was analysed by the BCA (Sigma, St. Louis, MO) procedure. The levels of Steroidogenic Acute regulatory protein (StAR) as well as of phosphorylation of Stat3 (that might reflect activation of the IL-6 signalling pathway) in adrenal homogenates were quantified by western blotting.

### Analyses

#### Quantitative real time PCR

Total RNA was isolated from frozen tibialis anterior, subcutaneous WAT and BAT using Tripure reagent (Roche Diagnostics Corporation, Indianapolis, IN) according to the manufacturer’s instructions. Analysis of *IL-6*, *Fgf21*, *Cs*, *Pdha-1*, *Ech1*, *Hadha*, *Bckdha*, *Bckdhb*, *CD36 and Fabp3* expression in tibialis anterior; *IL-6*, *Fgf21*, *Ech1*, *Hadha*, *CD36* and *Fabp3* expression in subcutaneous WAT; and *IL-6* and *Fgf21* expression in BAT were carried out in mice treated with vehicle or leptin.

Total RNA isolation from frozen hypothalamus was performed using Maxwell RSC simplyRNA tissue Kit and Maxwell RSC instrument (Promega), according to the manufacturer’s instructions. Reverse-transcription was performed using the iScript^TM^ cDNA Synthesis kit (BIO-RAD) following manufacturer’s instructions. Quantitative real time PCR (qPCR) was performed with iCycler Thermal Cycler (BioRad) using iTaq^th^ Universal SYBR® Green Supermix (BIO-RAD) to detect the amplification products. Analysis of *Trh*, *Crh*, *IL6*, *Socs 3*, and M*t1* expression in the response to stress experiment, and *Agrp*, *Crh*, *Pomc*, *Hcrt*, *Pmch* and *Npy* expression in the leptin experiment were carried out. Relative quantification of mRNA expression was normalized to the standard housekeeping gene *Gadph* and expressed as fold changes relative to the control group. Table 1 shows the primer sequences used for each gene.

**Table 1:** Primer sequences used.

Gene	Forward primer	Reverse primer
*Trh*	TCGTGCTAACTGGTATCCCC	CCCAAATCTCCCCTCTCTTC
*Crh*	CTTGAATTTCTTGCAGCCGGAG	GACTTCTGTTGAGGTTCCCCAG
*Socs3*	CAAGAACCTACGCATCCAGTG	CCAGCTTGAGTACACAGTCGAA
*Gapdh*	GGCAAATTCAACGGCACA	CGGAGATGATGACCCTTT
*Il6*	GCTTAATTACACATGTTCTCTGGGAAA	CAAGTGCATCATCGTTGTTCATAC
*Hcrt*	GCCGTCTCTACGAACTGTTGC	CGCTTTCCCAGAGTCAGGATA
*Agrp*	AGAGTTCCCAGGTCTAAGTCTG	GCGGTTCTGTGGATCTAGC
*Mt1*	TCACCAGATCTCGGAATGG	AAGAACCGGAATGAATCGC
*Npy*	ATGCTAGGTAACAAGCGAATGG	TGTCGCAGAGCGGAGTAGTAT
*Pmch*	GTCTGGCTGTAAAACCTTACCTC	CCTGAGCATGTCAAAATCTCTCC
*Pomc*	CGCAGAGGCGTGCGGAGGAAGA	TCCCTCTTGAACTCTAGGGGAAA
*Fgf21*	TTCTTTGCCAACAGCCAGAT	GTCCTCCAGCAGCAGTTCTC
*Hadha*	TGACGCTGGTTATCTTGCTG	ATCAGGGCCTTCGATTCTTT
*Cs*	CGGGAGGGCAGCAGTATCGG	ACCACCCTCATGGTCACTATGGATG
*Ech1*	TCGCTACTGCACTCAGGATG	AGCAGCCAAGCCCATATCTA
*CD36*	TTTGGAGTGGTAGTAAAAAGGGC	TGACATCAGGGACTCAGAGTAG
*Fabp3*	CATGAAGTCACTCGGTGTGG	TGCCATGAGTGAGAGTCAGG
*Bckdha*	AAGCCTCCTCTTCTCCGATGTG	GCAAAAGTCACCCTGGAATGC
*Bckdhb*	GTGCCCTGGATAACTCATTAGCC	CAAACTGGATTTCCGCAATAGC
*Pdha1*	GGGACGTCTGTTGAGAGAGC	TGTGTCCATGGTAGCGGTAA

#### Radioimmunoanalysis

Corticosterone RIA used ^125^I-corticosterone–carboxymethyloxime–tyrosine–methylester (MP Biomedicals, France) as the tracer, synthetic corticosterone (Sigma, Barcelona, Spain) as the standard, and an antibody raised in rabbits against corticosterone–carboxymethyloxime-BSA, which was kindly provided by Dr. G. Makara (Inst. Exp. Med., Budapest, Hungary) to Dr A. Armario. The characteristics of the antibody and the basic RIA procedure have been described previously [27]. All samples to be statistically compared were run in the same assay to avoid inter-assay variability. The intra-assay coefficient of variation was 7.8% and the sensitivity of the assays was 1 ng/ml.

#### Western blot

Western blots were carried out as follows. Tissues were homogenized in 50 mM Tris buffer (pH 7.5) containing 150 mM NaCl, 1 mM EDTA, 1 mM dithiothreitol, 10% glycerol, 50 mM Na-pyrophosphate, 50 mM sodium fluoride, 1 mM trisodium orthovanadate, 0.5 mM phenylmethylsulfonyl fluoride, and protease inhibitor cocktail (Sigma, St. Louis, MO) and centrifuged at 16.000 g for 15 min (4°C). Protein content of the soluble fraction was determined by the BCA method, and 50 μg were electrophoresed in a 4–12% SDS-PAGE gel and transferred to nitrocellulose membranes. Control samples were added to the gels for adjusting variations between gels and transference efficiency. The blot was stained with Ponceau-S and visually inspected for equal protein loading/transfer. The membranes were then blocked with 5% non-fat dried milk in Tween-TBS (TTBS) for 1 h. The blots were incubated with primary antibodies against pAkt (Ser473) (Cell Signaling Technology, USA; 1/1500 final dilution), Akt (Cell Signaling Technology, USA; 1/1500 final dilution), StAR (abcam, 1/5000 final dilution), and α-actinin (Cell Signaling Technology, USA; 1/1500 final dilution), P-Stat3 (Tyr 705) (Cell Signaling Technology, USA; 1/1500 final dilution) and Stat3(Cell Signaling Technology, USA; 1/1500final dilution) overnight at 4°C. After washing, appropriate secondary antibodies (anti-rabbit IgG-peroxidase conjugated [Calbiochem] or anti-mouse IgG-peroxidase conjugated [DAKO]) were used for 1 h at a dilution of 1/7500 and 1/5000 respectively. Blots were washed, incubated in commercial enhanced chemiluminescence reagents (ECL, Amersham Bioscience, UK). Protein bands were visualized using ChemiDoc XRS system (BioRad) and quantified using Quantity One software (version 4.6.3. Bio-Rad, UK).

#### Serum metabolites

Triglycerides, cholesterol (Linear Chemicals S.L), and glucose (Biomerieux S.A.) serum levels were measured by enzymatic colorimetric assays.

#### Statistical analysis

All data are presented as the mean ±SEM. Statistical package for Social Sciences (SPSS) was used. Data were analysed with Generalized Estimating equations for repeated measures, or ANOVA, with genotype, sex and/or treatment as main factors; if the interaction between factors was significant post-hoc analyses were carried out. When experimental set up precluded direct comparisons between sexes (such as separate western blots), statistical procedures were carried out separately.

## Results

### Energy expenditure

#### Absence of muscle IL-6 results in sexually dimorphic changes in basal metabolism

**Fig 2:**
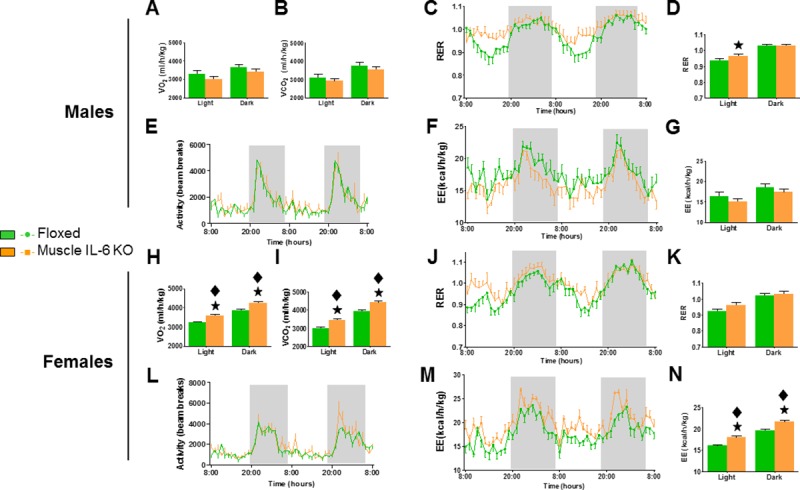
Indirect calorimetry measures of resting conditions (48 h). VO_2_, VCO_2_, RER, activity and EE means during the light and dark phase are shown in full and/or summarized as bar graphs. Muscle IL-6 deficiency in males (n = 7) results in significantly higher RER in the light phase (**C, D**) although no differences with floxed mice (n = 8) were noted in VO_2_ (**A**), VCO_2_, (**B**), activity (**E**) and EE (**F, G**). In females, significant differences between *muscle IL-6 KO* (n = 8) and floxed (n = 8) mice were noted in VO_2_ (**H**), VCO_2_ (**I**) and EE (**M, N**), but not in RER (**J, K**) and activity (**L**). ★ *p* at least < 0.05 *versus* floxed mice. ◆ *p* at least < 0.05 *versus* male mice.

Indirect calorimetry is often used in human and animal studies to determine total energy expenditure, and also to calculate the relative use of glucose and fatty acids (FAs) to support energy expenditure (respiratory exchange ratio, RER), assuming that contribution of protein oxidation is relatively constant and irrelevant [28]. As expected, in both sexes there was a strong circadian rhythmicity, with mice being more active, consuming more O_2_ and releasing more CO_2_ during the dark period (*P* < 0.05). Overall, deletion of IL-6 in muscles of male mice did not significantly affect oxygen consumption or CO_2_ release (Fig 2A and 2B) relative to floxed controls. However, their RER was significantly elevated, but only during the mid-part of the light period (Fig 2C and 2D). A reassessment of the oxygen and CO_2_ values during this period (between 11:00 and 16:00 h) revealed a tendency for elevated oxygen consumption in *muscle IL-6 KO* mice (*p* = 0.054) consistent with the observed elevation in RER values. In contrast, muscle IL-6 deficiency did not influence motor activity (Fig 2E) or energy expenditure (EE) over 48 h (Fig 2F) in either the light or dark phase (Fig 2G). No differences between genotypes were detected in food intake (data not shown).

On the contrary, in females, deficiency of muscle IL-6 produced significant (*p* < 0.001) increases of VO_2_ (Fig 2H) and CO_2_ release (Fig 2I) but did not influence the RER during the light or dark phases (Fig 2J and 2K). Also in contrast to males, EE measured over 48h (Fig 2M) revealed a significant elevation in *muscle IL-6 KO* female mice, in both light and dark phases (Fig 2N) (*p* <0.001). It is important to emphasize that this was not a general gender effect, but a specific effect of muscle IL-6 deficiency in female mice; accordingly, there were no differences between floxed male and female mice in VO_2_, CO_2_ and EE. No differences between genotypes were noticed in motor activity over 48h (Fig 2L) or in food and water consumption (data not shown).

#### Treadmill exercise induces changes in RER in muscle IL-6 KO males but not in females

**Fig 3:**
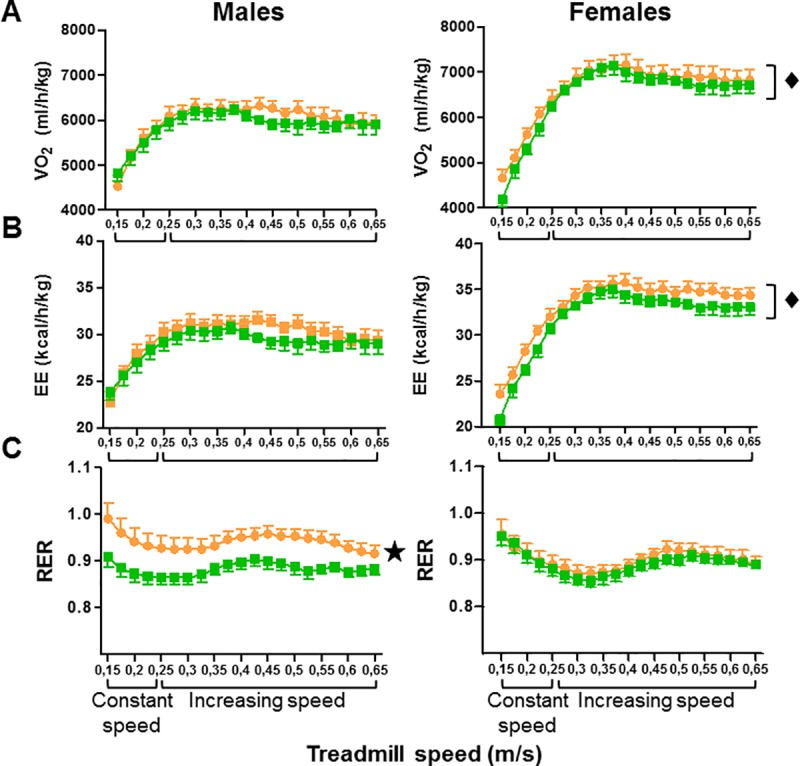
Indirect calorimetric parameters during acute exercise. The treadmill was initially set to a constant speed (0.15 m/s) for 2 min, increased at 0.2 m/s for 2 min, and then acceleration was increased by 0.05 m/s every two minutes. Although treadmill speed significantly affects VO_2_, EE and RER in both genders (**A-C**), only in males a significant effect of muscle IL-6 deficiency in RER (**C**) was noted. ★*p* < 0.05 *versus* floxed mice. ◆ *p* at least < 0.05 *versus* male mice.

During treadmill exercise, VO_2_ and EE increased for the first 6–8 minutes and then reached a plateau. Interestingly, in the response to exercise, female mice showed increased VO_2_ and EE compared to male mice, but no significant differences were found between *muscle IL-6 KO* and floxed controls of either sex (Fig 3A and 3B). However, male but not female *muscle IL-6 KO* mice exhibited significantly higher values of RER throughout the entire length of the exercise (Fig 3C).

#### Deficiency of muscle IL-6 determines a lower body core temperature in males during light phase

**Fig 4:**
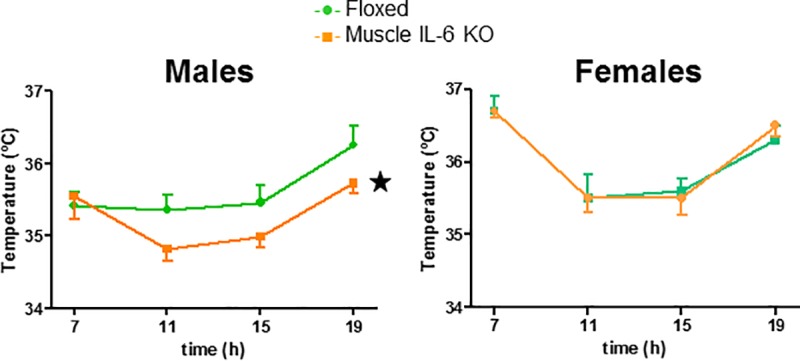
Core temperature in the light phase. Core temperature in floxed and *muscle-IL6 KO* male (n = 11/10), and female (n = 9/10) mice. Absence of muscle IL-6 determined a lower core temperature in male but not female mice in the light phase. ★ *p* at least < 0.05 *versus* floxed mice.

Body temperature cycle in the light phase showed significant differences in both sexes and genotypes. In male mice, body temperature was significantly different between floxed and *muscle IL-6 KO* mice; the latter showed a lower body temperature in the middle of the light phase and at 19:00 h, whereas the former showed a rather flat cycle at central light phase and higher body temperature at 19:00 h. Female showed a more prominent temperature cycle than male mice, but there were no significant differences between genotypes (Fig 4).

### Energy intake

#### Muscle IL-6 deficiency causes differences in female’s response to fasting

**Fig 5:**
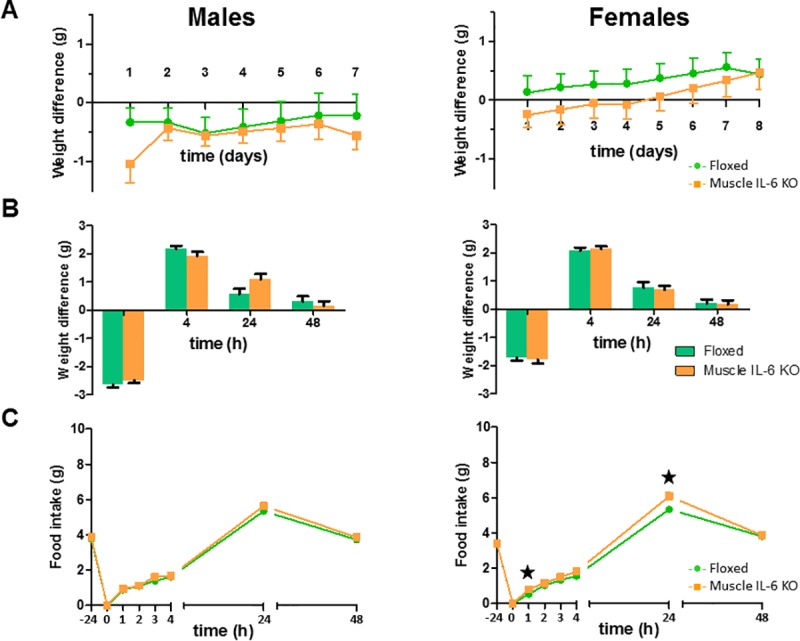
Body weight changes following fasting and refeeding. Adaptation to isolation measured by body weight gain in floxed and *muscle IL-6 KO* male (n = 13/12) and female (n = 14/14) mice (**A**). Female mice recovered somewhat faster but no effects of genotype were obvious. Following this period of acclimatization, the mice were subjected to fasting overnight and then food was allowed (refeeding) (**B, C**). Body weight changes following fasting and at 4, 24 and 48h after refeeding (**B**) and food intake at 1, 2, 3, 4, 24, and 48h after refeeding (**C**) are shown. The effect of muscle IL-6 deficiency was significant only for food intake and in some of the refeeding periods in female mice. ★ *p* at least < 0.05 *versus* floxed mice.

Since several experimental procedures relative to energy expenditure and intake were carried out with individually housed mice, we first evaluated the effect of isolation. Before isolation there were no significant weight differences between genotypes (~26.5 *vs* ~28.2 g for floxed and *muscle IL-6 KO* male mice; and ~20.02 *vs* ~20.52 g for floxed and *muscle IL-6 KO* female mice). During the isolation there were no significant overall effects of muscle IL-6 deficiency in both sexes. However in male mice there was a significant interaction between time of isolation and genotype (*p* <0.05) likely-because body weight loss during the first day was higher in *muscle IL-6 KO* mice. No differences in food intake between genotypes were observed either in the dynamics of intake or cumulative food intake (data not shown). After the 7–8 days of isolation, the mice weighed similarly (~26.3 *vs* ~27.6 g for floxed and *muscle IL-6 KO* male mice; and ~20.53 *vs* ~21.0 g for floxed and *muscle IL-6 KO* female mice), (Fig 5A).

Overnight fasting dramatically reduced body weight (>2.5 g; Fig 5B) and blood glucose levels (Table 2) in both genotypes and sexes; and upon refeeding, body weight was soon recovered (~2 g in the first 4 hours), returning to normal levels 24–48 hours later. However no obvious differences between genotypes were noted. At the end of this refeeding experiment, the mice weighed similarly again (~26.7 *vs* ~28.3 g for floxed and *muscle IL-6 KO* male mice; and ~21.8 *vs* ~22.2 g for floxed and *muscle IL-6 KO* female mice).

**Table 2:** Glucose values (mg/dl) in pre-fasting, immediately post-fasting and after 4h of refeeding * *p* at least <0.05 versus pre-fasting.

	MALES			FEMALES		
	Pre-fasting	Post-fasting	4h refeeding	Pre-fasting	Post-fasting	4h refeeding
Floxed	175.7 ±3.9	114.3±8.1 *	171.0 ±3.9	146.5 ± 4.2	105.0 ± 5.4*	165.6 ± 9.2
*MuscleIL-6 KO*	161.5 ± 5.7	129.9± 6.7*	175.1± 6.8	154.2 ± 4.3	114.1 ± 4.6 *	154.1 ± 5.2

Regarding food intake, when mice were allowed to eat after the overnight fasting, a clear and significant increase of food intake was observed compared to the previous food intake/24 hours (~5–6 g/day *vs* 3–4 g/day); this refeeding effect almost disappeared at 48 hours. Food intake during recovery time was significantly different between genotypes only in females, *muscle IL-6 KO* females showing significantly higher food intake than floxed females in the first hour and 24h after refeeding (Fig 5C).

#### Leptin treatment

Acclimatization to the injection procedure decreased body weight in both genotypes and sexes; this effect was in general more prominent in *muscle IL-6 KO* mice but it was not significant (data not shown). No differences in food intake were noted (data not shown).

**Fig 6:**
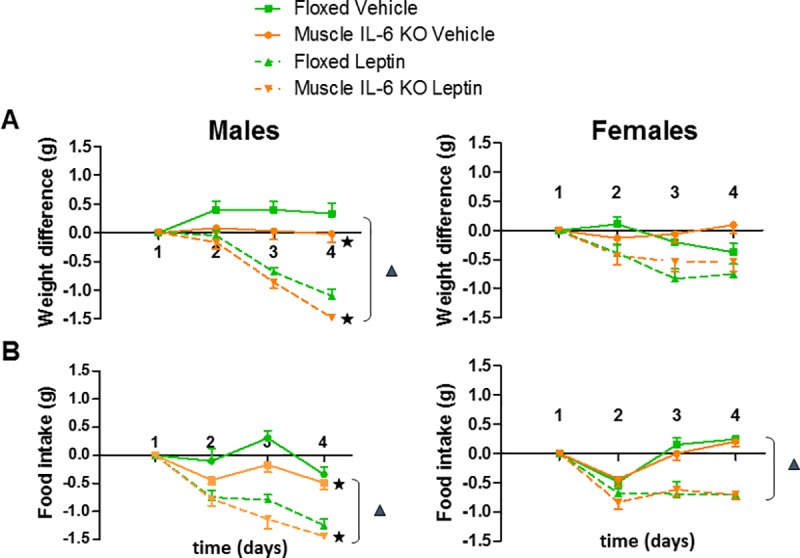
Effect of leptin treatment in body weight gain and food intake. Floxed and *muscle IL-6 KO* male (n = 13/12; **A**) and female (n = 14/14; **B**) mice were subjected to leptin treatment. Leptin was injected daily twice for three days and in the morning of the 4^th^ day, and 3 hours after the last injection animals were euthanized. Leptin decreased body weight gain (significantly only in males) and food intake; muscle IL-6 deficiency decreased body weight and food intake in male but not female mice, regardless of the specific treatment. ▲and ★ *p* at least < 0.05 *versus* vehicle and floxed mice, respectively.

As expected, leptin decreased body weight (*p* <0.05) and food intake (*p* <0.05). In males but not females, muscle IL-6 deficiency potentiated these responses, not only to leptin but also to vehicle injection (Fig 6A and 6B).

**Fig 7:**
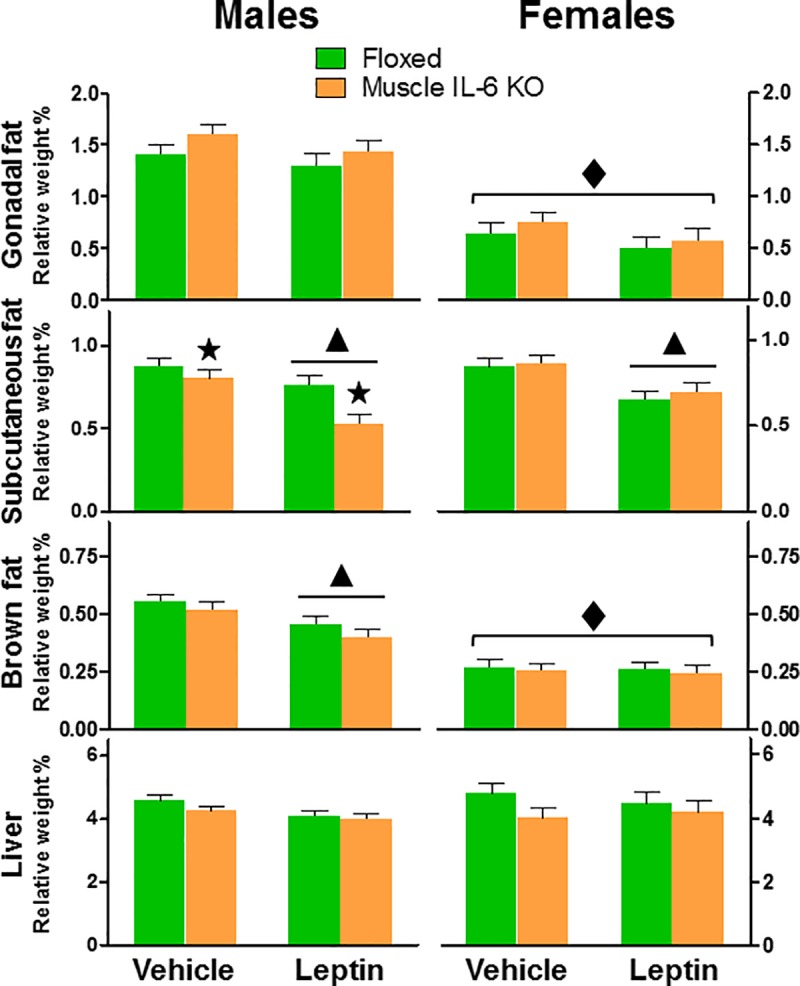
Effect of leptin treatment in WAT, BAT and liver weight. Gonadal, subcutaneous and brown fat depots and liver were weighed after leptin / vehicle treatment in males and females, and are expressed as the percentage of body weight. Female mice showed decreased depots of gonadal and brown fat *per se*. Additionally, subcutaneous fat depots were affected by leptin treatment in a gender specific manner, since in males, muscle IL-6 absence caused a higher loss of subcutaneous fat depots in response to leptin (34%). ▲and ★ *p* at least < 0.05 *versus* vehicle and floxed mice, respectively. ◆ *p* at least < 0.05 *versus* male mice.

Relative weight of subcutaneous fat was significantly decreased in both males and females of both genotypes with leptin treatment, but there was also a significant interaction between sex and genotype (*p*<0.05). A post-hoc analysis revealed that deficiency of muscle IL-6 significantly further decreased the relative weight of subcutaneous fat in males but not in females. Brown fat was also significantly decreased in males by leptin administration. No significant differences in relative liver weight were observed in either sex. On the other hand, female mice showed lower gonadal fat and brown fat than male mice, whereas no differences were observed for subcutaneous fat and liver (Fig 7).

**Fig 8:**
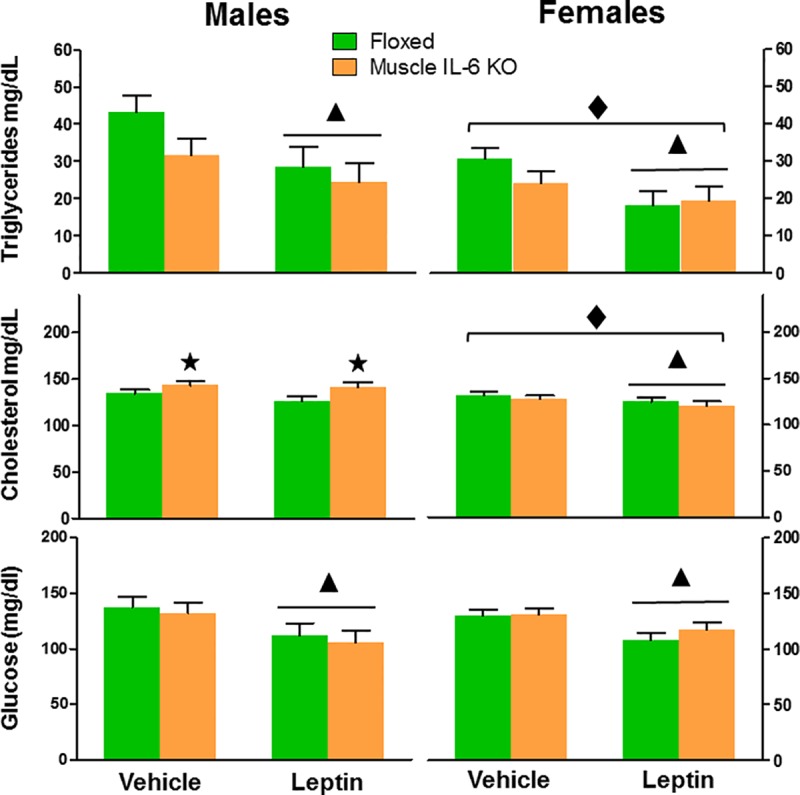
Analysis of plasma triglycerides, cholesterol and glucose levels after leptin treatment. Female mice showed decreased triglycerides and cholesterol levels *per se*. Leptin treatment significantly decreased triglycerides and glucose plasma levels in both genders, with no effect of muscle IL-6 deficiency. In contrast, leptin decreased cholesterol levels only in female mice, and muscle IL-6 deficiency increased serum cholesterol regardless of the treatment (vehicle or leptin) in male mice. ▲and ★ *p* at least < 0.05 *versus* vehicle and floxed mice, respectively. ◆ *p* at least < 0.05 *versus* male mice.

There was also a prominent effect of gender on serum levels of triglycerides and cholesterol (which were lower in female mice) but not of glucose. Glycaemia and triglycerides were decreased significantly by leptin in both sexes, with no clear-cut effects of muscle IL-6 deficiency (albeit a decreasing trend was observed for serum triglycerides, (*p* = 0.057). A different pattern emerged for serum cholesterol. There was an overall significant effect of leptin (*p*<0.05), but also a significant interaction between sex and genotype (*p*<0.001). A post-hoc analysis revealed that serum cholesterol levels were increased by muscle IL-6 deficiency in male mice regardless of leptin (Fig 8).

The anorexigenic effects of peripheral leptin treatment described above were reflected to some extent in its effect on hypothalamic neuropeptides known to influence body weight control. Thus, *Pomc* expression was significantly increased in male mice by leptin, and a non-significant trend for being increased by muscle IL-6 deficiency was observed (data not shown). The results for *Agrp* were less clear-cut and no significant differences were observed. A clear tendency for decreased *Crh* expression was also observed in *muscle IL-6 KO* male mice (*p* = 0.051). No significant differences in any of these hypothalamic factors were observed in female mice. Also, cortisol plasma levels were not affected by leptin treatment or muscle IL-6 deficiency (data not shown).

**Fig 9:**
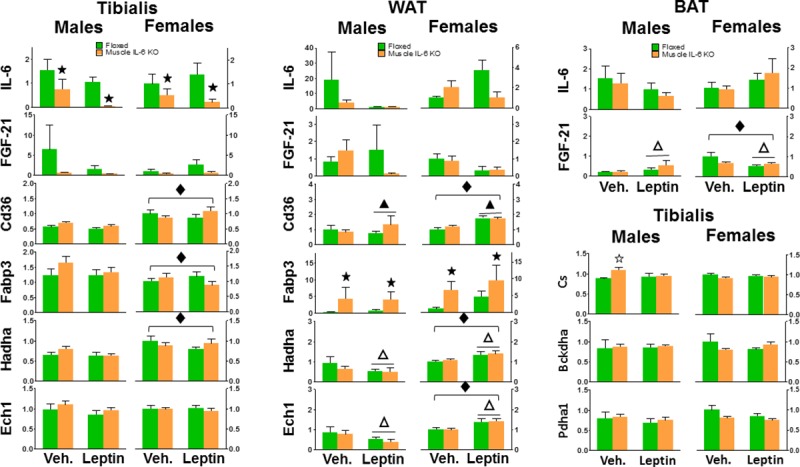
Gene expression in tibialis muscle, white adipose tissue and brown adipose tissue after leptin treatment. As expected, *muscle IL-6 KO* mice showed decreased IL-6 mRNA levels in muscle but not in other tissues. This muscle IL-6 deficiency did not affect the expression of the other genes in muscle; in contrast, it did affect that of Fabp3 in WAT. Sex was a clear factor influencing the expression of some of these genes, as was leptin injection. Interestingly, sex also modified the effect of leptin in WAT and BAT in some cases, inverting the effect of the hormone. ▲and ★ *p* at least < 0.05 *versus* vehicle and floxed mice, respectively. Δ denotes a significant interaction between sex and leptin *p* at least < 0.05. ◆ *p* at least < 0.05 *versus* male mice. ☆ denotes a significant interaction between sex and genotype *p* at least < 0.05.

The putative effects of muscle IL-6 deficiency and leptin treatment on several critical genes were also evaluated in selected peripheral tissues. In tibialis muscle, as expected, *IL-6* mRNA levels were significantly decreased in *muscle IL-6 KO* male and female mice. None of the other genes studied showed clear effects of either muscle IL-6 deficiency or leptin injection. Female mice had overall higher *CD36* and *Hadha* and lower *Fabp3* mRNA levels than male mice. There was a significant (p<0.05) interaction between sex and genotype for *Cs* expression, since *Cs* mRNA levels were increased by muscle IL-6 deficiency in male mice only, mostly in vehicle-treated animals. A similar trend was observed in *Fabp3* expression, but its higher variability precluded statistical significance (Fig 9 left).

In WAT, muscle IL-6 deficiency and leptin treatment had minor effects on the genes studied, with the notable exception of *Fabp3*, whose expression was increased by muscle IL-6 deficiency in both genders and regardless of leptin treatment. However leptin increased *CD36* expression, and there was a significant interaction between sex and leptin (*p* at least <0.05) in the case of *Hadha* and *Ech*, since leptin had a decreasing effect in male mice and the opposite was true in female mice. Female mice had overall higher *CD36*, *Hadha* and *Ech1* mRNA than male mice (Fig 9 middle).

In BAT, *IL-6* expression was again very low and thus of limited value. In contrast to the other two tissues, there was a significant interaction between sex and leptin in *Fgf-21* expression, since the hormone was increased in male mice and decreased in female mice (Fig 9 right).

### Insulin signalling pathway

**Fig 10:**
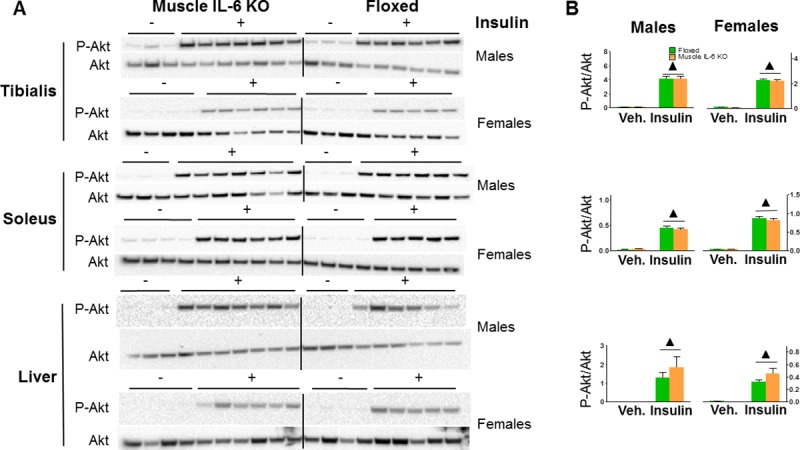
Deficiency of muscle IL-6 is not associated with changes in the muscle or liver insulin-signalling pathway. **A**: Representative western blot images of P-Akt/Akt in tibialis, soleus and liver from males and females, floxed and *muscle IL-6 KO* after 30 min of insulin administration (i.p. 1 UI/animal) or vehicle in 4 hours-starved mice. **B**: Quantification of the northern blots. ▲ *p* < 0.001 *versus* vehicle.

Deletion of muscle IL-6 did not modify the phosphorylation of Akt (Ser473) in muscle (tibialis or soleus) or liver of both genders (Fig 10A and 10B).

### Female stress response was affected by muscle IL-6 absence

**Fig 11:**
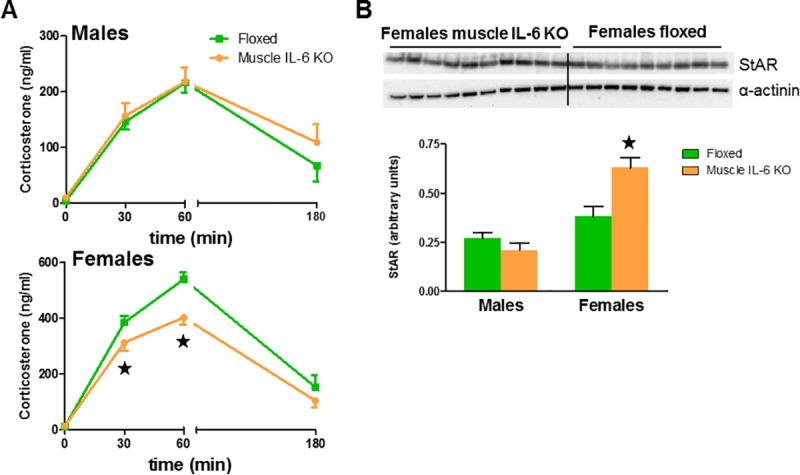
Deficiency of muscle IL-6 influences the response to restraint stress. **A**: Sixty min of restraint stress prominently increased serum corticosterone levels, which returned toward normal after 120 min of stress recovery. A sex-dependent effect of muscle IL-6-deficiency was present, since the stress response was lower in female but not male *muscle IL-6 KO* mice. **B**: Adrenal StAR levels in the adrenal glands were also evaluated by western blot. At the top the blot obtained for female mice is shown. At the bottom, the quantification carried out for both genders revealed increased adrenal StAR in females of *muscle IL-6 KO* after 120 min of stress recovery. ★ *p* at least < 0.05 *versus* floxed mice.

Circulating corticosterone levels of *muscle-IL-6 KO* females were significantly lower than those of floxed females after 30 and 60 min of restraint stress. Recovery levels at 120 min after stress finished were not different between genotypes. No differences in stress response and recovery between *muscle IL-6 KO* and floxed males were noted (Fig 11A). *Crh* levels measured following 1 h of stress plus 2 h of recovery indicated only a marginal effect of restraint stress (*p* = 0.054) in males (data not shown). In order to further establish putative differences in the HPA axis, adrenal weight and protein content were studied. No differences between genotypes in each sex were observed (data not shown). In contrast, significant higher StAR protein values were observed in *muscle-IL-6 KO* females than in floxed, with no differences in males (Fig 11B). Phosphorylation of Stat3 was not detectable in adrenal gland by our western blot procedure.

## Discussion

The present results support that muscle IL-6 has a relevant role in maintaining body energy homeostasis in a sex-dependent manner. This was obvious in RER results, *muscle IL-6 KO* male (but not female) mice had an increased RER in resting conditions during the central light phase, and consistent with this, they also had a lower core body temperature. The fact that RER values in *muscle IL-6 KO* male mice were closer to 1 during the light phase strongly suggests that they use more carbohydrates than fat as metabolic substrates. Previous studies with systemic IL-6 KO mice also showed decreased body temperature in males at room temperature during the light phase, and significantly higher RER and lower fat utilization rate [29–31]. Therefore, muscle IL-6 seems to account for most of the effects on RER of this cytokine. We also carried out indirect calorimetry analyses during treadmill exercise, and RER was again clearly increased in *muscle IL-6 KO* male mice only. This was also observed in 8-month-old systemic IL-6 KO mice, but not in 4-month-old mice [29]. In our study, the mice were young and definitely not obese and yet an increased RER was evident, which suggests that body weight *per se* is not the critical factor in this regard.

We observed an increased expression of Cs in tibialis anterior that eventually could indicate a higher TCA function, but since acetyl CoA may be produced from both carbohydrate and fat metabolism we cannot relate this easily to the RER results. While we were writing this manuscript the Pilegaard laboratory reported an RER increase in muscle-specific IL-6 KO males during 120 min of treadmill exercise [32]. They also observed a higher pyruvate dehydrogenase activity (PDH) at rest and after 60 min of exercise, and suggested that muscle IL-6 exerts an inhibitory action on skeletal muscle PDH and therefore on carbohydrate utilization, supporting the RER results. Discrepancies with other calorimetric studies with female systemic IL-6 KO mice [33] might be attributed to different WT genotype used (C57BL/6J) and/or other unknown reasons.

A clear sexual dimorphism was also observed regarding EE. Thus, *muscle IL-6 KO* female mice showed increased basal metabolism, in terms of EE levels, O_2_ production, and CO_2_ release whereas in males the opposite trend was observed. This increase in EE, in principle, might be related to the normal basal core temperature of *muscle IL-6 KO* female mice during the central light phase, in contrast to decreased values of *muscle IL-6 KO* male mice. Brown adipose tissue activity has been associated with non-shivering thermogenesis in mammals and *Fgf-21* secretion, which improves glycaemia and lipidaemia preventing some aspects of obesity and metabolic diseases [34]. Indeed, findings with transplanted BAT [35,36] suggest that BAT-derived IL-6 functions as a batokine *in vivo* regulating *Fgf-21* among other critical homeostatic factors. However no compensatory BAT *IL-6* or *Fgf-21* increased expression was found associated with muscle IL-6 deficiency.

Increased RER in *muscle IL-6 KO* mice, during resting conditions and during exercise, could also be due to effects of muscle IL-6 on the physical activity of the mice. However, no significant differences between genotypes were observed, ruling out a major role of muscle IL-6 controlling the activity of the animals. It is likely therefore that the effects previously reported in the hole-board test have more to do with exploration and/or anxiety than with activity *per se* [23]. However, the mice used in the present study have full backcrossing to a C57Bl/6 genetic background, which might also have a role in this apparent discrepancy.

Body weight and food consumption in basal conditions were not affected by muscle IL-6 deficiency in any of the experiments, which is consistent with our previous results [23] and with several studies using other transgenic mice [11,14,31]. Nevertheless, a role of muscle IL-6 might be relevant in other scenarios.

Overnight fasting dramatically reduced body weight, so a compensatory physiological response was to be expected. Once allowed to eat, the mice overfed the first day by ~50% relative to the previous days. By 48 hours, feeding was almost normal. Despite similar body weight changes in both genotypes and sexes, the dynamic of the refeeding was different, since *muscle IL-6 KO* mice tended to eat more during the first 24 h of refeeding, although this was significant only in females. This might have to do with the tendency of systemic IL-6 KO mice to become obese in the long term [37,38]. We previously [23] observed that *muscle IL-6 KO* female mice became obese when fed a high-fat diet, and the same tendency was present when fed a normal diet, despite food intake being normal.

Leptin is produced and secreted predominantly from adipose tissue, communicating energy storage to the brain [1–3]. As expected, leptin decreased food intake and body weight. Muscle IL-6 deficiency further decreased them, but only in males. It also decreased food intake and body weight in response to vehicle injections. Leptin stimulates fatty acid oxidation in muscle [39], producing a metabolic state in which energy must be obtained especially from subcutaneous fat. A leptin-specific interaction with IL-6 deficiency was present, since the decrease of subcutaneous fat was clearly higher in the leptin-treated male mice. This is consistent with previous results in *muscle IL-6 KO* male mice [23,40], but not with those in systemic IL-6 KO mice [11], again highlighting the relevance of the source of IL-6 in this regard.

Males and females may differ in their susceptibility to the obesity and the metabolic syndrome, and these differences could be associated with where they deposit fat [41]. Lipid deposition in subcutaneous fat allows efficient storage of maximal calories per unit volume of tissue [41]. Acute metabolic challenges, as well as genetically induced changes in fatty acid transporters, especially CD36, alter the rates of fatty acid transport and oxidation co-ordinately [42]; mice null for CD36 show significant increases in blood FA, TG and cholesterol [43]. Our results show that in male mice there is a lower expression of *CD36* in tibialis and WAT compared to female mice, suggesting a lower transport of fatty acids, perhaps accompanied by a lower capacity for beta oxidation, as suggested by the lower expression of *Hadha* (in both tissues) and *Ech1* (in WAT). These changes could be the cause of the increased cholesterol in plasma of male mice. This was more evident in *muscle IL-6 KO* male mice, probably due to alterations of the energy source rather than to changes in the expression of these genes.

*Fabp* expression in a given cell type seems to reflect its lipid-metabolizing capacity [44], and our results correlate muscle IL-6 deficiency with increased *Fabp3* expression in subcutaneous fat.

Peripheral leptin signals are detected and transduced, in part, by POMC and AGRP neurons in the arcuate nuclei of the hypothalamus [1]. However, only slight but not significant effects of IL-6 deficiency were observed in the expression of these genes. Whether this is due to the timing (several days after the first injection of leptin) or to something else needs further studies.

We observed that isolation (before the fasting/refeeding) and adaptation to daily saline injections (before leptin injections) seemed to have greater effects in *muscle IL-6 KO* mice. Thus, we reasoned that perhaps *muscle IL-6 KO* mice were more sensitive to stress. To ascertain this, a separate restraint stress experiment was carried out to evaluate the hypothalamus-pituitary-corticoadrenal axis (HPA). Female mice showed higher adrenal gland and stress circulating corticosterone levels than male mice, as expected [45,46]. *Muscle IL-6 KO* female mice responded less to restraint stress, in principle ruling out sensitivity to stress as the main reason underlying the differences in body weight during the acclimatization periods of the fasting/refeeding-leptin experiments. Corticosterone synthesis is predominately governed by mechanisms that determine StAR synthesis, activation, and degradation [47,48], and the immediate response to ACTH adrenal stimulation is achieved by the action of pre-existing StAR [49]. The increased StAR levels in *muscle IL-6 KO* female mice might therefore represent a compensatory response.

The role of IL-6 in insulin resistance is controversial [50,51]. We previously showed that muscle IL-6 deficiency did not significantly affect either the response to oral glucose administration or to insulin injection (OGTT and ITT, respectively) [23]. Here we analysed the response to insulin in several tissues by looking at Akt phosphorylation in fully backcrossed mice, and in accordance with the previous results, *muscle IL-6 KO* mice of both sexes responded similarly. Taken together, it is unlikely that muscle IL-6 is critical in insulin signalling in basal conditions.

In summary, our results highlight the critical regulatory role of muscle IL-6 in metabolic homeostasis of whole body in a gender-specific manner, not only during exercise but also in resting conditions and in a gender-specific manner. In addition, the data presented here indicate that muscle IL-6 absence may be relevant in situations in which the metabolic rate was changed, such as isolation, fasting, and leptin response. This work opens new approaches to study the role that the myokine IL-6 could play in the relationships between muscle and adipose tissue.

## Abbreviations

AgRP: agouti-related peptide
Akt: protein kinase B
Bckdha: branched chain keto acid dehydrogenase E1, alpha polypeptide
Bckdhb: branched chain keto acid dehydrogenase E1 subunit beta
CRH: corticotrophin releasing hormone
CD36: (thrombospondin receptor),(cluster of differentiation 36), also known as FAT (fatty acid translocase)
Cs: citrate synthase
Ech1: Enoyl-CoA Hydratase -1
EE: energy expenditure
FAO: fatty acid oxidation
Fabp3: fatty-acid-protein-binding-3
Fgf21: fibroblast growth factor 21
GABA: gamma-aminobutyric acid
GADPH: glyceraldehyde 3-phosphate dehydrogenase
Hadha: hydroxyacyl-CoA dehydrogenase/3-ketoacyl-CoA thiolase/enoyl-CoA hydratase (trifunctional protein), alpha subunit
Hcrt: hypocretin (orexin) neuropeptide precursor
HPA: Hypothalamus-Pituitary-Adrenal axis
IL-6: interleukin 6
Mt1: metallothionein 1
NPY: neuropeptide Y
Pdha-1: pyruvate dehydrogenase (lipoamide) alpha 1
Pmch: promelanin concentrating hormone
POMC: pro-opiomelanocortin
Stat3: signal transducer and activator of transcription 3
RER: respiratory exchange ratio
RIA: radioimmunoassay
StAR: steroidogenic acute regulatory protein
Socs 3: suppressor of cytokine signalling 3
TCA: tricarboxylic acid cycle
Trh: thyrotropin releasing hormone
